# The effect of subjective exercise experience on anxiety disorder in university freshmen: the chain-mediated role of self-efficacy and interpersonal relationship

**DOI:** 10.3389/fpsyg.2024.1292203

**Published:** 2024-02-21

**Authors:** Jun Xiang, Jia Gao, Yun Gao

**Affiliations:** ^1^School of Physical Education and Health, Zhaoqing University, Zhaoqing, China; ^2^Department of Physical Education, Shanghai Jiao Tong University, Shanghai, China

**Keywords:** subjective exercise experience, anxiety disorders, self-efficacy, human relations, mediating effect, freshman

## Abstract

**Background:**

Anxiety disorder is a significant concern in the context of mental health among university students. This study aimed to examine the impact of subjective exercise experience on anxiety disorder in freshmen and verify the mediating role of self-efficacy and interpersonal relationships between them.

**Methods:**

A total of 1,308 Chinese freshmen underwent an investigation using the Subjective Exercise Experience Scale (SEES), Generalized Anxiety Disorder Scale (GAD-7), General Self-Efficacy Scale (GSES), and Interpersonal Relationship Comprehensive Diagnostic Scale (IRIDS).

**Results:**

The outcomes of this study are as follows: (1) Boys exhibited marginally greater performance in physical activity assessments compared to girls while displaying somewhat lower scores than girls in measures of anxiety disorders, self-efficacy, and interpersonal relationship tests. Of these, 63.39% were diagnosed with mild anxiety, 51.73% were diagnosed with moderate anxiety, and 10% were diagnosed with severe anxiety; (2) The subjective exercise experience had a significant negative correlation with an anxiety disorder (*r* = −0.36, *p* < 0.01), and the subjective exercise experience had a direct negative impact on anxiety disorder (β = −0.112, *t* = −11.776, *p* < 0.01). Furthermore, subjective exercise experience positively predicted self-efficacy (β = 0.125, *t* = 13.236, *p* < 0.01) and interpersonal relationship (β = 0.395, *t* = 12.359, *p* < 0.01). Self-efficacy had a substantial impact on interpersonal relationships (β = 0.724, *t* = 12.172, *p* < 0.01) and anxiety disorders (β = −0.148, *t* = −8.387, *p* < 0.01). Interpersonal relationships had a significant positive predictive effect on anxiety disorder (β = −0.081, *t* = −10.441, *p* < 0.01); (3) Self-efficacy and interpersonal relationships were identified as important mediators between subjective exercise experience and anxiety disorder. The intermediary effect accounted for 18.84% of the total effect. Specifically, subjective exercise had a direct impact on anxiety disorders through self-efficacy mediators (2.90%), interpersonal mediators (1.45%), and self-efficacy and interpersonal chain mediators (14.49%).

**Conclusion:**

Subjective exercise experience has a significant positive predictive effect on university students’ self-efficacy, interpersonal relationships, and anxiety disorder. Moreover, self-efficacy and interpersonal interactions serve as intermediaries between subjective exercise experiences and anxiety disorders. These findings have immense importance in advancing the mental well-being of freshmen and serve as a theoretical foundation for formulating intervention strategies. However, the study had certain limitations, such as the specificity of the sample and the use of self-reported data. Further research could enhance the sample size and utilize various assessment techniques to validate these findings.

## Introduction

Anxiety is crucial in helping individuals adapt and respond effectively to environmental stimuli, particularly in hazardous circumstances (Aşçı, 2003). Individuals exhibit varying degrees of anxiety, a significant physiological characteristic inherent in the human body. It takes some anxiety to keep a person properly alert in a dangerous situation. However, suppose people have an inappropriate or excessive anxious response to external events. In that case, it easily leads to physical and psychological abnormalities in the human body, so in the long run, they may suffer from anxiety disorders (Szuhany Kristin and Simon, 2022; Singewald et al., 2023). Some studies believe that anxiety is a response to repressed sexual desire or aggressive impulse, which is about to break the psychological defense mechanism that maintains inner balance, so it is thought that anxiety may reflect inner contradictions and conflicts (Levitt, 2015; Freud, 2018; Perrotta, 2020). At present, China is in the stage of rapid economic development and continuous improvement of material level, but the number of people suffering from mental illness is increasing (Wedeman, 2012). The anxiety of university students is a hot topic that people have paid attention to in recent years.

With the rapid development of society, the expansion of university enrolment, the change in the education model, and other social factors, university students are under increasing pressure. When new university students leave their parents to study in different places for the first time, they often experience discomfort in their lifestyle and habits. This, along with poor handling of interpersonal relationships and other factors, contributes to the increasing prominence of mental health problems among them (Yimei et al., 2014). Thus, university students have gradually become a group with a high incidence of psychological crisis (Gang, 2010). Furthermore, University students suffer from high anxiety directly due to the pressure of homework, employment, exams, graduation papers, lack of communication between people, and other aspects (Wyatt and Oswalt, 2013). Compared with the general population, University students have higher psychological distress (Stallman, 2010). Studies have shown that autonomous motivation impacts the happiness of university students who engage in altruistic behaviors (Weinstein and Ryan, 2010). This study further supports the conclusions of domestic studies by Zhengxiong (2012), Zhang and Liu (2017), etc. That is, physical exercise plays an important role in reducing the anxiety level of university students. It is found that university students are facing increasing stress and anxiety, and physical exercise may be an effective intervention to reduce the level of anxiety. Therefore, this report aims to study the influence mechanism of subjective exercise experience on anxiety disorder of freshmen and analyze the mediating role of self-efficacy and interpersonal relationships to provide theoretical and empirical evidence for reducing anxiety disorder in freshmen.

## Literature review and research hypothesis

### Subjective exercise experience and anxiety disorder

Luo et al. (2023) have identified a significant association between subjective exercise experience and anxiety disorders, indicating a negative correlation between subjective exercise experience and anxiety levels. A survey was conducted on 500 university students, utilizing the Subjective Exercise Experience Scale and the Generalized Anxiety Disorder7. The findings indicated a negative correlation between the subjective exercise experience score and anxiety level, suggesting that a more favorable exercise experience was associated with reduced anxiety. This negative correlation may be because exercise can help individuals release tension, relieve stress, and enhance physical and mental resilience. Researchers also pointed out that exercise can promote dopamine release in the brain and enhance the psychological pleasure of individuals, thus reducing anxiety (Marques et al., 2021). Furthermore, researchers showed that physical exercise can promote blood circulation, improve brain oxygen supply, enhance cognitive function, and help individuals better deal with anxiety (Craft and Perna, 2004). There is a negative correlation between subjective exercise experience and anxiety disorder, implying that the better the subjective exercise experience, the lower the anxiety level. Therefore, Hypothesis 1 is proposed and subjective exercise experience has a positive predictive effect on anxiety disorder of freshmen.

### The mediating role of self-efficacy

The study examines the role of self-efficacy as a mediating mechanism. Self-efficacy serves as a crucial intermediary factor between an individual’s subjective exercise experience and the development of anxiety disorder. Several studies have demonstrated that the subjective exercise experience can alleviate anxiety by enhancing an individual’s self-efficacy (Liu, 2022; Wang et al., 2022). This study used the subjective exercise experience, self-efficacy, and Generalized Anxiety Disorder scales to investigate 300 University students. The findings suggest that there is a positive relationship between the subjective exercise experience and self-efficacy, but self-efficacy is inversely associated with anxiety. Additional researchers have indicated that self-efficacy can influence levels of anxiety through the following mechanisms. Individuals with high self-efficacy exhibit greater confidence in confronting obstacles and demands, enabling them to effectively manage anxious feelings and, therefore, diminish anxiety levels (Li and Xie, 2022). Furthermore, engaging in physical exercise, as a form of proactive action, can bolster personal self-efficacy, boost self-assurance, and foster enduring psychological resilience, thus aiding in the resistance to anxiety-inducing disruptions (Yang et al., 2023). Thus, we hypothesize that self-efficacy is a mediator between the subjective exercise experience and anxiety disorder in freshmen. In other words, the subjective exercise experience decreases anxiety by enhancing an individual’s self-efficacy. Therefore, Hypothesis 2 of this study is proposed, and self-efficacy plays a mediating role between subjective exercise experience and anxiety disorder of freshmen.

### The mediating role of interpersonal relationships

The second mediating mechanism in this study is the mediating role of interpersonal relationships. Some scholars have pointed out a positive correlation between subjective exercise experience and interpersonal relationships of university students (Kim et al., 2021). This study, 500 university students were investigated using a subjective exercise experience scale, interpersonal relationship evaluation scale, and anxiety disorder scale. The results showed that individuals with higher subjective exercise experience tend to have better interpersonal relationships because good interpersonal relationships positively reduce anxiety. First, individuals with good interpersonal relationships can receive adequate support and understanding, which helps relieve feelings of stress and anxiety (Costa et al., 2022). Second, feelings of intimacy and trust in interpersonal relationships can provide emotional security and reduce anxiety (Kural and Kovacs, 2022). Therefore, this study hypothesized that interpersonal relationships mediated the relationship between subjective exercise experience and anxiety disorder of freshmen; that is, subjective exercise experience alleviated anxiety by improving individual interpersonal relationships. Therefore, hypothesis 3 of this study is proposed: interpersonal relationship plays a mediating role between subjective exercise experience and anxiety disorder of freshmen.

### The chain-mediating effect of self-efficacy and interpersonal relationship

Self-efficacy and interpersonal relationships may play a chain mediating role between subjective exercise experience and anxiety disorder. Researchers showed that self-efficacy refers to an individual’s belief and ability assessment of whether he can effectively complete a task (Sherafati and Mahmoudi, 2023). A strong sense of self-efficacy can motivate individuals to respond positively to duress and challenges by increasing their awareness of their capabilities. Research has established a positive correlation between self-efficacy and subjective exercise experience (Wang et al., 2022). Consequently, those with greater levels of subjective exercise experience are more likely to have higher self-efficacy. Other researchers have noted that interpersonal connections significantly influence an individual’s self-efficacy. The endorsement and assistance of others within a healthy interpersonal connection can bolster individuals’ self-assurance and belief in their abilities and self-efficacy (Zhang and Li, 2022). Moreover, intimacy and support in interpersonal relationships can also provide emotional satisfaction and security, further enhancing self-efficacy (Byun and Kim, 2022). Therefore, this study hypothesized that self-efficacy and interpersonal relationships play a chain mediating role between subjective exercise experience and anxiety disorder of freshmen, and subjective exercise experience can improve interpersonal relationships and reduce anxiety by improving individual self-efficacy. Therefore, hypothesis 4 of this study is proposed: self-efficacy and interpersonal relationship mediate between subjective exercise experience and anxiety disorder of freshmen.

**Figure 1:**
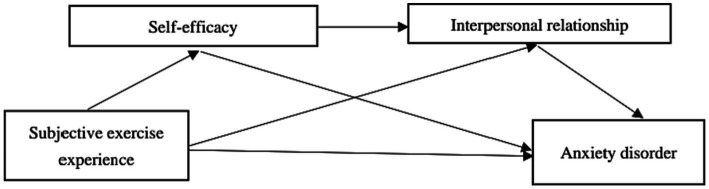
Research paradigm diagram.

In summary, this study established the concept of research framework, as depicted in Figure 1: (1) To examine the predictive effect of subjective exercise experience on anxiety disorder of freshmen; (2) To investigate the mediating role of self-efficacy between subjective exercise experience and anxiety disorder of freshmen; (3) To investigate the mediating role of interpersonal relationships between subjective exercise experience and anxiety disorder of freshmen; (4) Finally, to examine the chain mediating effect of self-efficacy and interpersonal relationship on subjective exercise experience and anxiety disorder of freshmen.

## Research object method

### Research object

The subjective exercise experience scale, anxiety disorder scale, self-efficacy scale, and interpersonal relationship scale were used to conduct a random sampling survey of Chinese freshmen. Economic level and other factors were considered to make the samples more representative. According to the current administrative division of China, four universities (24 in total) were randomly selected in North China, Northeast China, East China, Central South China, Southwest China, and Northwest China, and two classes (48 in total) were randomly selected from the freshmen of each university. A total of 1,423 questionnaires were distributed. The participants underwent testing during their physical education class, with the primary examiners consisting of qualified physical education instructors and psychology students. The test received approval from school administrators, teachers, and students, and all surveys were filled out within 10 min. The questionnaire was also administered in a group setting, and the subjects were instructed to fill out the questionnaire using standardized instructions. The inclusion criteria for the subjects before the questionnaire was distributed were: (1) physically and mentally healthy; (2) freshmen; (3) no major diseases; and (4) those who had participated in physical exercise and subjects who did not meet the above criteria were excluded from the study. After the questionnaires were recovered, they were excluded according to the following criteria: (1) missing data, (2) inconsistent responses, (3) those who had no physical activity, and (4) those who had limited physical activity. After collation, 1,308 valid questionnaires were recovered in this study (115 subjects were excluded due to missing data on the main variables and inclusion criteria), with a recovery rate of 92%. Participants ranged in age from 17 to 23 years old (*M*age = 19, *SD*age = 1.15), including 478 males and 830 females. There was no significant difference between the variables of sex and grade. Furthermore, the Institutional Review Committee of Zhaoqing University supported and approved the study. All freshmen who participated in the survey signed an informed consent form. The informed consent for this study describes the purpose and process of the study, the methods used, and other contents, and also includes information such as confidentiality guarantee, voluntary participation principle, and contact information of researchers; the variables such as gender, class, and region of the subjects were also controlled.

### Research method

#### Psychometric method

##### Subjective exercise experience measurement

The Subjective Exercise Experience Scale (SEES) was developed by McAuley and Courneya in 1994 to measure the effects of exercise stimulation on exercisers’ positive emotions, negative emotions, and subjective physical expenditure (McAuley and Courneya, 1994). In 2004, the Subjective Exercise Experience Scale was revised in the Evaluation Manual of Commonly Used Psychological Scales in Sports Science (Zhang and Mao, 2004), so the revised Subjective Exercise Experience Scale was adopted in this study. In previous studies (Zhonghua, 2021; Zhang, 2022), the subjective exercise experience scale was used to measure the subjective exercise experience of Chinese University students and had good reliability and validity. The 12-item Subjective Exercise Experience Scale is a seven-level Likert scale that contains three dimensions: positive well-being (e.g., “I feel great”), psychological annoyance (e.g., “I feel terrible”), and fatigue (e.g., “I feel exhausted”). The total score of the subjective exercise experience scale is 84 points, and the total score of the three dimensions is 24 points. The answer options are designed from “often inconsistent” to “very consistent,” with a total of 7 options (1 means very inconsistent; 2 indicates no match; 3 indicates that the comparison is inconsistent; 4 means compromise; 5 indicates more consistent; 6: Yes. 7 indicates a strong match). Among them, the positive happiness subscale includes the scores of 1, 4, 7, 10, and 4 items. The higher the score, the stronger the positive happiness experience. The psychological distress subscale includes the scores of four items: 2, 5, 8, and 11. The higher the score, the more serious the distress level. The fatigue subscale includes the total score of four items (3, 6, 9, and 12). The higher the score, the more severe the fatigue degree. The sum of the scores from the three aspects yields the overall score of the subjective exercise experience scale. According to Peilin-Pan (2021), there is a positive correlation between the total score on the subjective exercise experience scale and the level of subjective exercise experience. In other words, as the total score increases, the level of subjective exercise experience also increases. Conversely, a lower total score indicates a lower level of subjective exercise experience. Cronbach’s α coefficient of the scale in this study was 0.79, showing good reliability. Additionally, the confirmatory factor analysis showed a strong fitting index, suggesting that the questionnaire was valid in this study.

##### Generalized anxiety disorder measurement

Generalized Anxiety Disorder 7 was developed in 2006 by Spitzer RL, Kroenke K, and Williams JBW (Spitzer et al., 2006) and used to screen and assess the severity of the subjects’ anxiety states. In 2010, Xiaoyan et al. translated and revised the scale to form the Chinese version of GAD-7 (Xiaoyan et al., 2010). In previous studies (Xiaowen, 2022), the Generalized Anxiety Disorder Scale (GAD-7) has been used to measure generalized anxiety disorder in Chinese University students, with good reliability and validity. The Generalized Anxiety Disorder Scale comprises seven items, including question 1, which assesses the presence of feelings of nervousness, anxiety, or discomfort. The scale utilizes a 4-tier scoring system (0–3 points), with a range of values from 0 to 21 points. The total value is obtained by summing the scores of the seven items; a higher score indicates more severe symptoms. The classification of anxiety levels is as follows: 0–4 is categorized as no anxiety, 5–9 as mild anxiety, 10–14 as moderate anxiety, and ≥ 15 as severe anxiety. The Cronbach’s α coefficient of this scale in this study was 0.86, indicating high internal consistency. Additionally, the confirmatory factor analysis yielded a satisfactory fitting index, suggesting that the questionnaire showed strong reliability and validity in this study.

##### General self-efficacy measurement

The General Self-Efficacy Scale (GSES) was developed in 1981 by Professor Ralf Schwarzer, a distinguished clinical and health psychologist at the Free University of Berlin, Germany, and his colleagues. The GSES has been translated into at least 25 languages and is extensively utilized globally (Schwarzer et al., 1999). In 1995, Jianxin Zhang and Schwarzer introduced the Chinese version of GSES to first-year University students in Hong Kong. Since then, the Chinese version of GSES has been found to possess favorable reliability and validity (Zhang and Schwarzer, 1995). In 2001, Caikang et al. conducted another study on the reliability and validity of the Chinese version of GSES, and the results also confirmed the relevant findings of Schwarzer et al. that the Chinese version of GSES has higher reliability and prediction validity (Caikang et al., 2001). In previous studies (Chengjian et al., 2023), the general self-efficacy scale was used to measure the self-efficacy of Chinese University students, and it has good reliability and validity. The general self-efficacy scale consists of 10 items involving the self-confidence of individuals when they encounter setbacks or difficulties. The four-point Likert scale is adopted (1 point for “completely incorrect,” 2 points for “somewhat correct,” 3 points for “mostly correct,” 4 points for “completely correct”). The GSES is a single-dimensional scale (for example, question 1: “If I try my best, I can always solve the problem”); there is no subscale, so only the total is counted. The total score of the scale is equal to the sum of the scores of 10 items divided by 10, and the higher the score, the higher the self-efficacy. In this study, the Cronbach’sα coefficient of this scale was 0.89, and the fitting index of confirmatory factor analysis was good, indicating that the questionnaire had good reliability and validity in this study.

##### Interpersonal relationship comprehensive diagnostic measurement

The Interpersonal Relationship Comprehensive Diagnostic Scale, developed by Richang of Beijing Normal University in 1996, is a tool used to assess individuals’ interpersonal distress (Richang, 1996). It comprises four factors: distress in conversation, distress in communication, distress in dealing with people and things, and distress in dealing with the opposite sex. The scale has a total of 28 items; each item has “yes” and “no” two options (for example, question 1: “about their troubles”); according to 1 and 0 points, the full score is 28 points. The total score of the scale is calculated by adding the scores of all components. The individual’s total score ranges from 0 to 8 points, indicating a lower anxiety level in interpersonal communication. The individual income score ranges from 9 to 14 points, indicating a moderate level of communication difficulty for individuals. The aggregate score for individual income ranges from 15 to 28 points, signifying significant difficulty in individual communication. In previous studies (Wenjing, 2019), the Interpersonal Relationship Comprehensive Diagnostic Scale was used to measure the interpersonal relationships of Chinese University students, and it has good reliability and validity. In this study, the Cronbach’sα coefficient of this scale was 0.92, and the fitting index of confirmatory factor analysis was good, indicating that the questionnaire had good reliability and validity in this study.

##### Mathematical statistics

This study carefully examined the collected questionnaire to identify and remove any incorrect data. The statistical data analysis was then conducted using SPSS21.0 software and the SPSS macro program Process plug-in developed by Hayes. First, the SPSS21.0 program was utilized to perform descriptive statistics, including calculating means and standard deviations. A difference test was also conducted to analyze the demographic information, subjective exercise experience, anxiety disorder, self-efficacy, and interpersonal connection test data. The significance level for this test was set at *p* < 0.05. Second, SPSS21.0 software used common latent variables to assess common method bias. Third, SPSS21.0 was used to examine the Pearson bivariate relationship among freshmen’s subjective exercise experience, anxiety disorder, self-efficacy, and interpersonal relationships. Fourthly, the PROCESS plug-in model 6 and Bootstrap (5,000 times) sampling technique were used to examine the independent mediating effect between self-efficacy and interpersonal relationship and the chain mediating effect between subjective exercise experience and anxiety disorder. In this study, p < 0.05 was set as a statistical result with significance.

## Research results

### Descriptive statistics of subjective exercise experience, anxiety disorders, self-efficacy, and interpersonal relationships

Table 1 Statistical results showed that subjective exercise experience and interpersonal relationships had no statistical significance in gender difference analysis (*p* > 0.05). In contrast, anxiety disorder and self-efficacy had statistical significance in gender difference analysis (*p* < 0.05). Subjective exercise experience, anxiety disorder, self-efficacy, and interpersonal relationships were statistically significant in age difference analysis (*p* < 0.05). Boys scored slightly higher than girls on physical activity tests but slightly lower on anxiety disorders, self-efficacy, and interpersonal relationships. According to the test results of the sample survey, 658 detected mild anxiety, occupying 50.31% of the total sample; 537 detected moderate anxiety, occupying 41.05% of the total sample; and 113 detected severe anxiety, occupying 8.64% of the total sample, indicating that freshmen do have varying degrees of anxiety, which contributes to further research in this paper. Moreover, the four variables showed certain regularity in different statistical calibers, which is helpful to understand further the influence degree and mutual relationship of subjective exercise experience, self-efficacy, and interpersonal relationship on anxiety disorder.

**Table 1:** Descriptive statistics of subjective exercise experience, anxiety disorder, self-efficacy, and interpersonal relationship test results (*x* ± SD).

Sex	N/people	Subjective exercise experience	Anxiety disorder	Self-efficacy	Interpersonal relationship
Male	478	45.30 ± 10.31	9.14 ± 3.55	28.62 ± 5.76	46.49 ± 14.24
Female	830	44.98 ± 9.72	10.34 ± 3.84	26.95 ± 4.98	47.66 ± 11.65
Totality	1,038	45.10 ± 9.93	9.90 ± 3.78	27.56 ± 5.34	47.23 ± 12.66
Sex difference (T/p)		0.35/0.57	0.27/0.00	7.98/0.00	24.32/0.11
Age difference (F/p)		2.48/0.00	0.80/0.71	2.19/0.00	1.85/0.00

*p* < 0.001 represents significant difference between sex and age.

### Common method deviation test

Using questionnaires to collect data may have the risk of common methodology bias (Blanchett and Israelsen, 2007). Therefore, the Harman single-factor test was used for statistical control before data analysis, that is, unrotated principal component factor analysis for all variables (Zhou and Long, 2004). The results showed that seven factors with eigenvalues greater than one were extracted from the results of unrotated exploratory factor analysis, and the variance explained by the first factor was 29.76%, less than the critical value of 40% (Rahimi et al., 2023). This indicates that there are no factors that can explain most of the variation in this study, so there is no serious common method bias in the data of this study, which meets the conditions for further testing of the chain mediation effect.

### Correlation analysis of subjective exercise experience, anxiety disorder, self-efficacy, and interpersonal relationship

Table 2 demonstrates a clear positive correlation between subjective exercise experience and both self-efficacy and interpersonal relationships. Additionally, there is a strong negative correlation between subjective exercise experience and anxiety disorder. There was a favorable correlation between self-efficacy and interpersonal interactions, and a negative correlation between self-efficacy and anxiety disorder. A strong inverse association was seen between interpersonal relationships and anxiety disorders. The study found substantial correlations between the variables of subjective exercise experience, anxiety disorder, self-efficacy, and interpersonal interaction. These relationships support the subsequent hypothesis testing and provide a solid foundation for investigating the mediation effect in this study.

**Table 2:** Correlation analysis and statistics of subjective exercise experience, anxiety disorder, self-efficacy, and interpersonal relationship.

Variable quantity	Subjective exercise experience	Anxiety disorder	Self-efficacy	Interpersonal relationship
Subjective exercise experience	1			
Anxiety disorder	−0.36**	1		
Self-efficacy	0.56**	−0.356**	1	
Interpersonal relationship	0.59**	−0.42**	0.29**	1

*N* = 1,308. **p < 0.001. The same below.

### Testing the mediating effect of self-efficacy and interpersonal relationship

**Figure 2:**
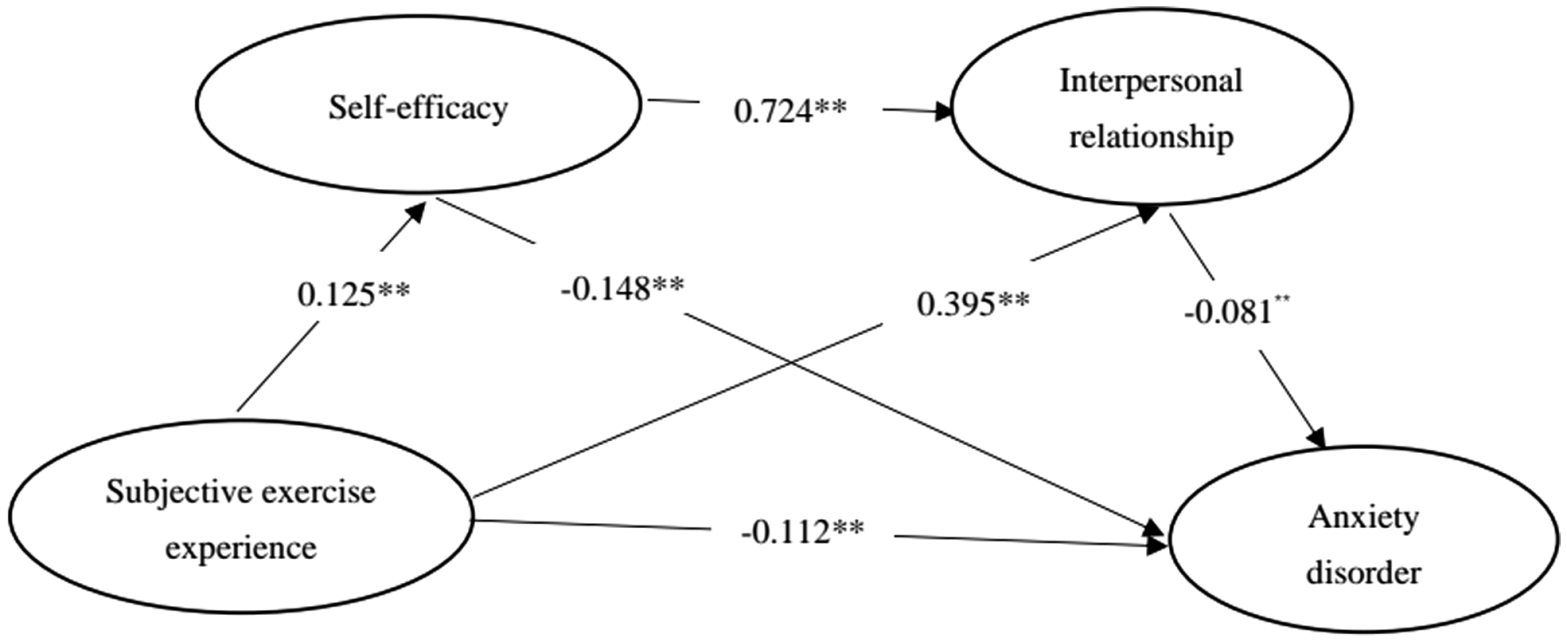
The chain mediation model of self-efficacy and interpersonal relationship between subjective exercise experience and anxiety disorder.

Subjective exercise experience was taken as the independent variable, self-efficacy and interpersonal relationships were the mediating variable, and anxiety disorder was the dependent variable. The SPSS macro plug-in PROCESS, developed by Hayes, was adopted (Hayes, 2013). Model 6, as per the Templates, was chosen. The 95% confidence interval was calculated using the deviation-corrected non-parametric percentile Bootstrap test method, which involved repeated sampling 5,000 times. This method was used to examine the impacts of the chain mediation model (Little et al., 2007; Zhonglin et al., 2022). Hence, based on the findings shown in Table 3 and the path coefficient values illustrated in Figure 2, the overall impact of the subjective exercise experience on the anxiety disorder of freshmen was determined to be 0.138 (*t* = 14.118, *p* < 0.001). The path coefficient of subjective exercise experience on anxiety disorder of freshmen was −0.112 (*t* = −11.776, *p* < 0.001). The path coefficient of subjective exercise experience on self-efficacy was 0.125 (*t* = 13.236, *p* < 0.001). The path coefficient of self-efficacy in anxiety disorder was −0.148 (*t* = −8.387, *p* < 0.001). The path coefficient of subjective exercise experience on interpersonal relationships was 0.395 (*t* = 12.359, *p* < 0.001). The path coefficient of interpersonal relationships for anxiety disorder was −0.081 (*t* = −10.441, *p* < 0.001). The path coefficient of self-efficacy in interpersonal relationships was 0.724 (*t* = 12.172, *p* < 0.001), and all path coefficients reached the significance level (*p* < 0.001). The results confirm the hypothesis H1.

**Table 3:** Regression analysis of the chain-mediated model between subjective exercise experience and anxiety disorders.

Variable quantity	Self-efficacy		Interpersonal relationship		Anxiety disorder		Aggregate effect	
	β	t	β	t	β	t	β	t
Subjective exercise experience	0.125	13.236**	0.395	12.359**	−0.112	−11.776**	0.138	14.118**
Self-efficacy			0.724	12.172**	−0.148	−8.387**		
Interpersonal relationship					−0.081	−10.441**		
*R* ^2^	0.123		0.181		0.278		0.132	
*F*	121.833		143.767		166.995		199.308	

*p < 0.05, **p < 0.001.

After conducting a standardized effect size and significance test on the relationship between subjective exercise experience and freshman anxiety disorder, it was observed (as shown in Table 4) that the Bootstrap of the 95% confidence interval for the total indirect effects of subjective exercise experience and freshman anxiety disorder does not encompass the value of 0. The results indicate that “self-efficacy” and “interpersonal relationship” play a crucial role in moderating the relationship between subjective exercise experience and anxiety disorder. The intermediary effect consists of three indirect effects: (1) For the indirect effect produced by the path of “subjective exercise experience” → “self-efficacy” → “anxiety disorder.” the Bootstrap of 95% confidence interval does not include 0, indicating that self-efficacy plays a significant mediating role between subjective exercise experience and anxiety disorder (standardized value = −0.004, accounting for 2.90% of the total effect). This result verifies Hypothesis H2. (2) The indirect effect produced by the path of “subjective exercise experience” → “interpersonal relationship” → “anxiety disorder,” the Bootstrap 95% confidence interval does not include 0, indicating that interpersonal relationship plays a significant mediating role between subjective exercise experience and anxiety disorder (standardized value = −0.002, accounting for 1.45% of the total effect). The result verifies Hypothesis H3. (3) The indirect effect produced by the path of “subjective exercise experience” → “self-efficacy” → “interpersonal relationship” → “anxiety disorder,” the Bootstrap of 95% confidence interval does not contain 0, indicating that self-efficacy and interpersonal relationship play a significant chain mediating role between subjective exercise experience and anxiety disorder (standardized value =0.020, Accounting for 14.49% of the total effect), this result verifies hypothesis H4.

**Table 4:** Test of chain-mediated effects of self-efficacy and interpersonal relationship on subjective exercise experience and anxiety disorder.

Effect type	Effect size	Boot SE	Bootstrap95% CI		Effect ratio
			Floor	Upper limit	
Total effect	0.138	0.01	0.119	0.158	100%
Direct effect	0.112	0.01	0.093	0.13	81.16%
Subjective exercise experience – self-efficacy – Anxiety disorder	−0.004	0.003	−0.01	−0.003	2.90%
Subjective exercise experience – Interpersonal relationship – Anxiety disorder	−0.002	0.001	−0.004	−0.001	1.45%
Subjective exercise experience – self-efficacy – Interpersonal relationship – Anxiety disorder	0.02	0.004	0.024	0.041	14.49%
Total indirect effect	0.026	0.007	0.015	0.04	18.84%

## Discussion

### Subjective exercise experience and anxiety disorders

This study showed that subjective exercise experience had a significant negative effect on freshmen’s anxiety disorders (β = −0.112, *p* < 0.001), i.e., the better the freshmen’s subjective exercise experience, the less their anxiety disorders. Hypothesis 1 was verified, and this result is consistent with the findings of previous studies. A strong inverse relationship exists between the subjective perception of exercise and the level of anxiety. During exercise, people’s perception of their emotional state influences their anxiety levels. Individuals having a more positive and enjoyable exercise experience improves their mental health and reduces the likelihood of anxiety disorders (Akhdan and Aminatun, 2022; Takiguchi et al., 2023; Wong et al., 2023). Research indicates that physical activity can serve as a coping mechanism for stress. This is because it triggers the release of a morphine-like substance in the brain, which helps reduce stress by diverting attention away from potentially stressful situations. Consequently, it also alleviates anxiety by redirecting the individual’s focus (Sran et al., 2021). Encouraging and promoting active engagement in physical activity among freshmen can offer a pragmatic approach to relieving symptoms of anxiety.

Furthermore, according to the data from the research tests in this study, freshmen do have varying degrees of anxiety. According to the psychological theory of anxiety, anxiety is an intense distress and frustration of the individual’s feelings and experiences, is inexplicable rather than a specific event or object in their own life or work feel uneasy. The term “anxiety” describes a strong and lasting emotional response that occurs when a person perceives a potentially harmful stimulus, either internal or external, as a threat to their self-worth and believes they are unable to handle it. This response leads to physiological and behavioral changes (Barlow, 1991). Studies have demonstrated that an excessive amount of anxiety has detrimental impacts on human behavior and personality. Anxiety can result in retreat, excessive adherence to norms, and dread, hence impeding academic and personality growth (Ambrose, 2020). For first-year students, the unsettling circumstances of separating from their parents for the initial time to pursue education in a new location, together with adjustments in lifestyle and habits, can contribute to a rise in mental health issues. Over time, individuals will encounter several pressures and difficulties, rendering them more susceptible to increased anxiety (Yimei et al., 2014). Therefore, it is particularly important to address the problem of anxiety disorders among freshmen, which is consistent with the results of the data testing in this paper. Based on the findings of this study, it is evident that targeted intervention for anxiety disorders among freshmen can be achieved through physical exercise. This intervention is crucial and pressing, and enhancing the quality of the exercise experience may serve as a potential solution to alleviate anxiety among freshmen. Engaging in exercise as an enjoyable and rewarding activity can serve as a helpful approach to managing anxiety issues among freshmen.

### The mediating role of self-efficacy

This study showed that self-efficacy mediated the relationship between subjective exercise experience and anxiety disorders (β = −0.004, *p* < 0.001), i.e., the effect of subjective exercise experience on freshmen’s anxiety disorders was realized through the mediating variable of self-efficacy. Hypothesis 2 was verified. This is consistent with the results of previous studies. A significant positive correlation exists between individuals’ subjective exercise experience and self-efficacy, and subjective exercise experience positively predicts self-efficacy (Tian and Shi, 2022). During exercise, individuals’ subjective evaluation of their abilities affect their self-efficacy and anxiety levels, and by increasing the level of college students’ subjective exercise experience, their confidence in their abilities can be enhanced, their self-efficacy can be increased, and their anxiety can be reduced (Luo et al., 2023). First, according to Bandura’s self-efficacy theory, negative emotional states and imagined experiences reduce an individual’s sense of self-efficacy (Bandura, 1977). This reduced belief leads individuals to display more avoidant and withdrawn attitudes and behaviors in future social interactions (Fan et al., 2010). Second, anxiety theory posits that individuals experience a sense of self-inefficacy when they encounter anxiety-inducing events that may be unpleasant. People with high self-efficacy can effectively deal with or stop a negative occurrence from happening to themselves. Additionally, having a strong belief in one’s abilities can reduce feelings of anxiety (Kashdan and Roberts, 2004; DaLomba et al., 2023). Low self-evaluation and lack of social skills are the primary factors contributing to anxiety in college students. Specifically, individuals with lower self-evaluation tend to have higher levels of social anxiety (Purdon et al., 2001). It can be seen that freshmen who just stepped into a new environment to study and live often encounter a lot of things that do not go well and do not adapt to; their self-efficacy is reduced, which later leads to an increase in their anxiety. This is consistent with the results of this study. Finally, scholars point out that self-efficacy is a positive psychological indicator that can stimulate people’s subjective initiative and help them overcome difficulties. Engaging in positive emotions during exercise leads to a higher level of exhaustion, which enhances exercisers’ ability to develop a positive mindset, self-efficacy, and exercise goals. The subjective exercise experience aims to elicit a range of emotional and physiological responses following physical activity. Individuals with a heightened level of subjective exercise experience are capable of experiencing positive emotions during exercise and possess an optimistic mindset for problem-solving in challenging situations (Beibei-Huang, 2023). The recent research literature, both nationally and globally, confirms that self-efficacy acts as a mediator between subjective exercise experience and anxiety disorders, as found in this study. These findings highlight the significance of enhancing an individual’s self-efficacy by increasing their subjective exercise experience to decrease anxiety disorders.

### The mediating role of interpersonal relationships

This study also found that the connection between subjective exercise experience and anxiety disorders was influenced by interpersonal relationships (β = −0.002, *p* < 0.001); this suggests that the impact of subjective exercise experience on anxiety disorders in freshmen was manifested through interpersonal relationships, which acted as another mediating factor. Thus, Hypothesis 3 was verified and is consistent with the results of previous studies. First of all, sports have a positive impact on interpersonal relationships (Wenjing, 2020). Engaging in sports activities regularly for an extended period promotes the development of positive interpersonal relationships among students. Students who have a habit of physical exercise have significantly better interpersonal relationships than those who do not regularly participate in physical exercise (Ren et al., 2021; Zhang et al., 2022). Sports allow students to communicate and interact, creating a relaxed atmosphere and reducing pressure. This facilitates more relaxed exchange and communication among classmates, helping to avoid the discomfort that may arise from poor interpersonal relationships. And help students overcome their timid and shy nature, establish self-confidence, improve social interaction, and form more intimate interpersonal relationships (Wenjing, 2020; Feiyi et al., 2021). Then, studies also point out that the status of interpersonal relationships is closely related to mental health (Zhang et al., 2019; Cheng et al., 2020), and poor interpersonal relationships may lead to depression, anxiety, and even suicidal tendencies (Chunfen et al., 2020; Di et al., 2022). Because freshman year is the interface and excessive period between high school and college, freshmen will need a certain period of adaptation in interpersonal relationships, life, and learning when facing a new college environment (Bowman et al., 2019). They leave the original familiar living environment, and college life creates greater learning and life pressure, which is prone to anxiety, which can lead to interpersonal distress (Zhang et al., 2019). According to personality development theory, during the college years, the primary objective of individual personality development is to cultivate intimacy and prevent feelings of loneliness. Developing and sustaining positive interpersonal connections is essential for college students, as interpersonal interactions significantly contribute to socialization and social adjustment (McWhirter, 1997; Reis, 2001; Nurmi, 2004). Anxiety arises from the discrepancy between the self and the ideal self. To alleviate anxiety, it is necessary to establish a genuine interpersonal connection between the real and ideal selves and to accept the real self to develop a practical sense of purpose for the future (Lundy and Drouin, 2016; Siegel et al., 2018). Regular sports activity can relieve psychological pressure, form good psychological quality, and improve mental health ability (Yue et al., 2019). It can be seen that the findings of scholars have shown that interpersonal relationships are closely linked between subjective exercise experience and anxiety disorders and the importance of good interpersonal relationships for the self-efficacy and psychological health of individuals. Contemporary college students can enhance their interpersonal interactions and alleviate college-related anxiety by engaging in physical exercise. College students should adapt their principles and cultivate positive interpersonal relationships, regardless of the challenges they may face in their future academic and personal endeavors.

### The chain mediating effect of self-efficacy and interpersonal relationship

According to this study, it was found that self-efficacy and interpersonal interactions act as a chain mediator between subjective exercise experience and anxiety disorders (β = 0.020, *p* < 0.001). The study found that the impact of an individual’s personal exercise experience on the development of anxiety disorder in freshmen was observed through the sequential mediation of self-efficacy and interpersonal interactions. Thus, Hypothesis 4 has been confirmed. These findings are consistent with the outcomes of prior research. First, subjective exercise experience can increase individuals’ self-efficacy (Wang et al., 2022). When individuals feel their progress and achievement through subjective exercise experience, their confidence in their ability will increase, thus enhancing self-efficacy (Yue, 2021). Furthermore, individuals’ self-efficacy also impacts the quality of interpersonal relationships, and individuals with higher self-efficacy are more likely to establish and maintain good interpersonal relationships (Byun and Kim, 2022). This means that individuals’ confidence in their abilities affect how they interact with others and their ability to communicate, affecting the quality of interpersonal relationships. Self-efficacy refers to an individual’s belief in their ability to effectively participate in and sustain social interactions required for interpersonal relationships. It is a specific manifestation of self-efficacy in the social realm. The quality of interpersonal relationships is strongly linked to an individual’s self-efficacy. Positive interpersonal relationships offer emotional support and acknowledgment, hence boosting an individual’s self-confidence. Finally, researchers observed that strong interpersonal interactions can offer emotional support and safety, increasing self-efficacy and decreasing anxiety (Chenran, 2021; Commodari et al., 2022). Because the quality of interpersonal relationships impacts an individual’s self-efficacy and anxiety levels, they develop greater confidence in their abilities when they receive support and recognition from others (Huang et al., 2023). And by strengthening the interpersonal and social support of college students, their self-efficacy can be improved, and their anxiety levels can be reduced (Wang et al., 2023). We found a close association between subjective exercise experience, interpersonal relationships, self-efficacy, and anxiety disorders, consistent with the results of this study.

Engaging in subjective exercise enhances interpersonal interactions and diminishes anxiety by auguring an individual’s self-efficacy. The findings of this study underscore the significance of self-efficacy and interpersonal ties in assisting freshmen in managing anxiety disorders. Subjective exercise experiences were found to have a direct impact on anxiety disorders, with self-efficacy and interpersonal interactions acting as intermediary factors in this association. Subjective exercise experience encompasses the exercisers’ subjective perception of positive and negative emotional states and physiological exertion following physical activity. This factor significantly impacts students’ engagement in physical education and is crucial in enhancing teaching quality. Hence, it is recommended that, in the future, the mental well-being of freshmen can be addressed by promoting favorable subjective exercise encounters and cultivating positive interpersonal connections, thereby mitigating the anxiety experienced by freshmen during their academic tenure.

## Research deficiencies and prospects

First, this study investigated the factors that contribute to anxiety disorders in freshmen and suggested that subjective exercise experiences and interpersonal relationships play a role in reducing anxiety and enhancing self-efficacy. This study holds significant theoretical significance in comprehending the underlying factors contributing to anxiety among freshmen in college. Additionally, it catalyzes developing strategies to avoid and intervene in anxiety disorders within this specific population. The current results enhance the previous knowledge by offering empirical evidence for the influence of subjective exercise experience on anxiety control. This underscores the significance of considering subjective emotions and experiences when comprehending the intricate interplay between physical activity and mental well-being. Additionally, it emphasizes the potential advantages of using subjective evaluations in future studies on exercise therapies for anxiety disorders. The findings of this study have practical, helpful implications for mental health professionals and educational institutions to recognize the impact of subjective exercise experiences on anxiety disorders. Targeted interventions can be developed to implement programs that promote positive exercise experiences, such as providing a variety of enjoyable physical activities and creating supportive environments, which may help to reduce anxiety levels in incoming students.

Second, schools should prioritize the extensive development of students, with particular emphasis on physical activity. Schools should organize comprehensive orientation programs to familiarize new students with campus resources, academic expectations, and available support services. They should also facilitate communication between new and experienced students or faculty members who can provide guidance and support during the transition. It is important to clearly and consistently communicate information about course requirements and available resources. Additionally, schools should ensure that students who may be experiencing anxiety or other mental health challenges have access to confidential counseling services, fitness programs, nutritious food choices, and recreational activities. These initiatives aim to promote healthy lifestyles among students. Colleges and universities should enhance efforts to prevent and manage mental health issues among college students while improving their social interaction skills. This will help prevent or reduce interpersonal relationship difficulties among students and promote their overall physical and mental well-being. To implement intervention programs and tools effectively, physical education teachers must communicate clearly and develop strategies to alleviate students’ anxiety in school sports. Physical education teachers need to create a respectful and friendly environment. Providing individualized support through targeted feedback and recognition of achievement increases student confidence. Strive to maintain a harmonious equilibrium between academic and athletic obligations, prioritize enjoyment, and address issues related to performance anxiety. Utilizing diverse science-based teaching approaches and resources fosters student engagement, creating a conducive environment for children to enjoy physical activity while efficiently addressing anxiety. Parents need to establish and maintain effective communication channels with freshmen to listen to their problems and offer assistance attentively. Occasionally reinforce the significance of self-care to first-year students, including regular engagement in physical activity, consumption of a well-rounded diet, and obtaining sufficient rest. Facilitate and support students in accessing the various resources, both on and off campus, that are available to them for academic and emotional assistance. Individual freshmen should manage their time and prioritize their tasks to ensure they can complete their academic and personal responsibilities without overwhelming themselves. Simultaneously, they should promptly seek assistance during difficult periods, establish attainable objectives to foster self-assurance and a feeling of accomplishment, engage in regular physical activity, integrate regular exercise, nutritious eating, optimistic thinking, and relaxation methods into their daily routines, and actively partake in school-sponsored activities to cultivate a sense of teamwork and acquire new abilities.

However, this study has certain limitations. First, the cross-sectional design of this study does not allow for the inference of a causal relationship, and the use of experimental interventions or follow-up studies could be considered in the future to explore mechanisms further. Conversely, longitudinal studies would provide valuable insights into the temporal dynamics of this relationship. Second, we used a single survey instrument to assess which common methodological variations may be biased. It is recommended that multiple assessment tools be used to collect data in the future. As our study focused on freshmen with a sample from a specific university and limited generalizability, self-reported data may be subjectively biased, and objective assessment measures could be considered to improve reliability. Broadening the scope of the investigation is recommended. Additional research is needed to determine if the findings may be applied to other populations. Furthermore, future research endeavors could explore the fundamental processes through which subjective exercise experiences impact anxiety levels. Further research is recommended to investigate the impact of psychosocial elements, including social support, peer support, physical self-esteem, and other related issues. Future research endeavors may incorporate a range of evaluation instruments and research methodologies, expand the sample size, apply objective assessment measures, and consider supplementary factors to enhance our comprehension and management of anxiety disorders in freshmen.

## Conclusion

Subjective exercise experience and interpersonal relationships were not statistically significant in gender differences, while anxiety disorder and self-efficacy were statistically significant in gender differences. Moreover, age differences were statistically significant in subjective exercise experience, anxiety disorder, self-efficacy, and interpersonal relationships. Boys scored slightly higher than girls on tests of physical activity but slightly lower on tests of anxiety disorders, self-efficacy, and relationships. There are different degrees of anxiety among freshmen in our country.

Subjective exercise experience is important in predicting self-efficacy, interpersonal relationships, and anxiety disorders among freshmen. Positive subjective exercise experiences help to enhance individuals’ sense of self-efficacy and make them more confident in their abilities. Good interpersonal relationships can also enhance an individual’s sense of self-efficacy.

The subjective exercise experience reduced anxiety in freshmen by increasing their self-efficacy and boosting their interpersonal interactions. Further, subjective exercise experience predicted the self-efficacy, interpersonal relationships, and anxiety disorders of freshmen.

## Data availability statement

The data that support the findings of this study are available from the corresponding author upon reasonable request.

## Ethics statement

The studies involving humans were approved by Committee of the Academic Committee of the School of Physical Education and Health, Zhaoqing University. The studies were conducted in accordance with the local legislation and institutional requirements. The participants provided their written informed consent to participate in this study.

## Author contributions

JX: Writing – original draft, Writing – review & editing. JG: Data curation, Investigation, Writing – review & editing. YG: Writing – review & editing.
